# Semiconductor Work and the Risk of Spontaneous Abortion: A Systematic Review and Meta-Analysis

**DOI:** 10.3390/ijerph16234626

**Published:** 2019-11-21

**Authors:** Kyungsik Kim, Ho Kyung Sung, Kwan Lee, Sue K. Park

**Affiliations:** 1 Department of Preventive Medicine, Seoul National University College of Medicine, Seoul 03080, Korea; kks6235@snu.ac.kr; 2 Department of Biomedical Science, Seoul National University Graduate School, Seoul 03080, Korea; 3 Cancer Research Institute, Seoul National University College of Medicine, Seoul 03080, Korea; 4 National Emergency Medical Center, National Medical Center, Seoul 04564, Korea; hokyungsung@nmc.or.kr; 5 Department of Preventive Medicine, Dongguk University College of Medicine, Gyeongju 38066, Korea; kwaniya@dongguk.ac.kr

**Keywords:** semiconductor, spontaneous abortion, meta-analysis, photolithography

## Abstract

(1) Background: In the semiconductor industry, female workers were identified as having an increased risk of spontaneous abortion (SA). To date, the association between semiconductor work and SA is controversial. We aimed to assess the association between semiconductor work and specific processes and SA, in the semiconductor industry. (2) Methods: A literature search was conducted using databases such as PubMed, Embase, Cochrane library, and other core databases, from the date of inception of these databases to 31 July 2019. Studies that identified SA risk in female workers in the semiconductor industry were included. (3) Results: We identified 529 studies, of which six studies were included in the meta-analysis. During 1980–1993, the risk of SA in fabrication (Fab) workers was significantly higher than non-Fab workers (RR, 1.29; 95% CI, 1.05–1.57). Photolithography workers had a higher SA risk than non-process and office workers (RR, 1.41; 95% CI, 1.13–1.77). (4) Conclusion: Meta-analysis indicates a statistically significant association between Fab-work and SA. Specific process and chemical exposure meta-analyses need to be interpreted carefully considering bias. Because of the rapid change in the semiconductor industry, it is necessary to conduct an elaborate cohort study taking into consideration the current working environment.

## 1. Introduction

The semiconductor industry began in the 1960s [1], and Korea built the foundation for the semiconductor industry in the 1970s [2]. The semiconductor industry has the second-highest proportion of female workers among the twelve main industries in Korea [3]. Due to employment promotion among women in the semiconductor industry, their exposure to chemicals in the occupational environment has become more frequent [4]. According to Korea Employment Information Service, nearly 107,000 workers are involved in the Korean semiconductor industry, which accounts for 0.7% of the entire working population [5]. Female workers in the semiconductor industry account for about 30.8% of all workers, more than any other manufacturing industry [3]. There is an increase in the proportion of female workers in the semiconductor industry, and most of them are of childbearing age [6]. They could be exposed to various chemicals causing reproductive toxicity such as lead, arsenic and arsenical compounds, tetrachloroethylene, trichloroethylene, ionizing radiations, and organic solvents [7]. As a result, concerns about female workers and their offspring have been steadily increasing [8,9].

Several studies discuss the health outcomes, including reproductive toxicity, according to chemical exposure in female workers [10,11,12]. Until recently, several studies have reported on workers’ health problems such as reproductive toxicity, cancer, etc. but the causality between occupational diseases and specific chemical exposure or semiconductor work has not yet been identified [13,14,15]. Occupational exposure on the reproductive system could alter workers’ sex hormone levels. Alteration in sex hormone levels leads to various reproductive problems including menstrual disorders, premature menopause, ovarian dysfunction, and reduced fertility. Chemical exposure in pregnancy can affect fetal development by directly or indirectly disrupting maternal, placenta, or fetal membrane function. Although there are differences depending on the exposure period, duration, and dosage, abortion occurs due to early pregnancy exposure [16]. Among reproductive toxicity, spontaneous abortion (SA) presents its reproductive hazard earlier than other outcomes (stillbirth or congenital anomalies). It is important to assess reproductive toxicity in the semiconductor industry [17]. Since most reproductive toxicity studies on semiconductor workers were conducted mainly on SA [13], it is necessary to conduct a systematic review and meta-analysis of SA.

In the 1980s, the first study reported that the risk of SA in workers exposed to semiconductor process was relatively higher than in unexposed workers [18]. During the 1980s and 1990s, the distribution of work of the Irish electronics industry workers showed that several female workers worked as operators and could be exposed to chemicals [19]. In 2014, a narrative review was published on the health outcomes of semiconductor workers [20]. However, their SA risk was not evaluated based on systematic review or meta-analysis, and several studies were reported from before same cohort source. Therefore, systematic reviews and meta-analyses need to consider it. In addition, in 2017, a study based on Korean semiconductor workers was reported in which the SA risk in fabrication (Fab) and assembly workers was compared to office workers according to pregnancy time. There was a statistically significant difference when the pregnancy year was prior to 2008, but not significant after 2009 [21].

The semiconductor industry technology has been changing rapidly, and the hazardous materials used in the process are changing frequently [22]. Solvents exposure data from Taiwan’s plants indicate that materials such as butanone, methanol, and acetone were used until the early 1980s and are no longer used [23]. In addition, few studies have considered the current working environment. Thus, we aimed to conduct a systematic review and meta-analysis of the semiconductor work and SA. Furthermore, in our study, we aimed to identify the risk of SA due to specific processes and chemicals in the semiconductor industry.

## 2. Materials and Methods

### 2.1. Search Strategy

During the process, we followed the Preferred Reporting Items for Systematic Reviews and Meta-Analyses (PRISMA) guideline. Literature search was conducted using databases including PubMed, Embase, Cochrane library, KoreaMed, Research Information Sharing Service (RISS), National Digital Science Library (NDSL), and Korean Medical Database (KMBASE) from their inception to 31 July 2019. As search terms, we used various combinations: (1) (“occupational disease” OR “occupational exposure” OR “spontaneous abortion”) AND “semiconductor”; and (2) “semiconductor” AND “spontaneous abortion”. To find more relevant studies, we manually searched through the references of the studies. All studies from the manual search were included in the database search. Endnote X8 (Thomson Reuters, New York, NY, USA) software was used to manage the studies during the process. Two independent reviewers went over the relevant studies.

### 2.2. Selection Criteria

Studies based on the association between semiconductor work and SA were discussed in the systematic review and meta-analysis. During the database search, we excluded studies which did not relate with the objective by reading the title and abstract. For further review, we scrutinized the full-text of each study before including it in this review. We considered semiconductor workers of any age and ethnicity as participants. Semiconductor manufacturing process can be roughly divided into wafer manufacturing, fabrication process, and assembly (packaging) process. Among them, the fabrication process is the process of processing wafers to form and integrate semiconductor circuits. Oxidation, deposition, cleaning, photoresist coating, ion implantation, development, etching, etc. are repeated several times. There were divided into four main groups: (1) photolithography and etching; (2) diffusion and ion implantation; (3) deposition-epitaxial or chemical vapor deposition (CVD); and (4) metallization-sputtering and evaporation [24]. If each document could be classified in detail, photolithography and diffusion processes were separately defined as specific processes. The process and exposed chemicals in each study are based on the assessment from industrial hygienist by personnel interviews and records. In the meta-analysis, we conducted a study based on the following criteria: (1) fabrication work exposure; (2) specific process exposure (identified as photolithography or EGE (Ethylene Glycol Ether) exposure); and (3) EGE exposure (identified as EGE chemical exposure). Female workers or wives of male workers in general semiconductor work, Fab, specific-process, or circuit-board process in the semiconductor industry were included as the exposed population. For comparison, we considered the general population, non-Fab or workers not involved in processing such as office or administrative staff. As outcome, we considered SA, which was identified through structured interview and medical records. Cohort and case-control studies that identified the odds ratio (OR) or relative risk (RR) were included in the meta-analysis. If there were studies from the same source, the more recent or more informative study was selected. The following studies were excluded: in vivo and in vitro studies, measurement of the validity and reliability, chemical exposure assessment, disease diagnosis using semiconductor technology, studies that did not provide 95% confidence intervals (95% CI) or effect sizes, and studies that were not published in English.

### 2.3. Quality Assessment

To assess quality, we used the Newcastle–Ottawa Scale (NOS) for observational studies, especially cohort studies and case-control studies [25]. To estimate the sum of the NOS score, two independent reviewers evaluated and scored each study. Reviewers compared the calculated scores with each other, and, when the scores were not identical, the quality assessment was reevaluated by another reviewer. The NOS comprises three parts: selection, comparability, and outcome or exposure. In the cohort study, representativeness of the exposed cohort, the selection method of the non-exposed cohort, and the ascertainment of the exposure were included in the selection component. In the case-control study, the definition of the case group, the representativeness of the case group, the selection of the control group, and the definition of the control group were included. For comparability, the comparability of the cohort (case-control) in design or analysis is included. In the case of outcome, the cohort studies include information on the assessment outcome, the adequacy of the follow-up period, and the follow-up rate, while the case-control studies include information on the assessment of exposure, the equivalence method of case and control groups, and the non-response rate [25]. Both case-control studies and cohort studies with NOS scores over five points were included in the meta-analysis. According to the quality assessment, we classified studies with over six points as high quality and those with 2–5 points as low quality.

### 2.4. Data Extraction

Two reviewers extracted data from the included studies. The information was the name of the author, publication year, working period, study design, remarks, study location, the total number of participants, number of SA, exposure and comparison, OR, RR, and 95% CI.

### 2.5. Statistical Analysis

In the meta-analysis, we used the random effect model, which is the variance effect model, to calculate the integrated summary statistics of the measures reported in the literature. In studies where the 95% CI was not provided, it was calculated using the appropriate formula and included in the meta-analysis [26]. According to the studies, only Fab workers or specific-process workers were considered as exposed groups. If there were more than two documents with the same outcome variable, a meta-analysis was applied, and the summary statistics are presented. If there were two or more studies based on the same study design after quality assessment, we conducted stratification analysis [27]. Statistical heterogeneity among studies was evaluated with the Cochran Q and I^2^ statistics [28,29]. In the case of the Cochran Q test, *p*-value < 0.1 was observed when the homogeneity was violated. I^2^ statistics test is advantageous since it is not affected by the number of studies compared to Cochran Q. Usually, 25–49%, 50–74%, and over 75% indicate low, intermediate, and high heterogeneity, respectively [29]. We considered stratification analysis or a sensitivity analysis [27], when there was heterogeneity because of the difference in the criteria of the study subject, criteria of the exposed group, method of measuring the result variable, or research design. Publication bias was evaluated using Egger and Begg tests, in which a *p*-value < 0.05 was considered representative of statistically significant publication bias [30,31]. If there was publication bias, we used trim and fill method or sensitivity analysis to adjust it [27,32]. Statistical analyses were conducted using STATA SE software version 14 (StataCorp, College Station, TX, USA).

## 3. Results

**Figure 1:**
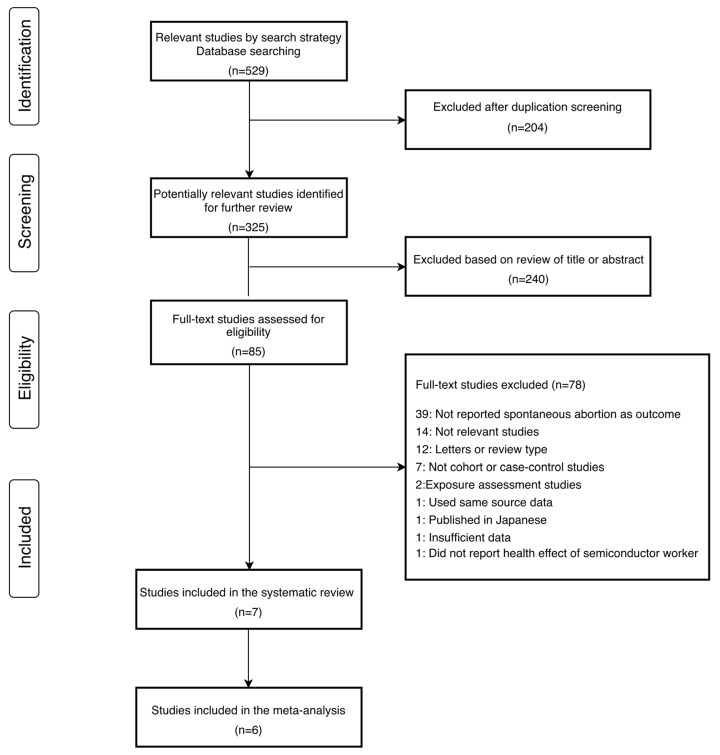
Progression on the selection of studies for the meta-analysis.

Figure 1 shows the progression in the selection of studies for the meta-analysis. According to the search strategy, 529 studies were identified using the database search. Of these, 204 studies were duplicates and thus excluded. After reviewing the titles and abstracts of 325 studies, we excluded 240 studies. The remaining 85 studies were considered eligible for the meta-analysis, and these articles were reviewed in detail. There were seven studies included in the systematic review, among which six were included in the meta-analysis. Their main characteristics are summarized in Tables S1 and S2. Among the six studies in meta-analysis, four were cohort studies [18,33,34,35], and two were case-control studies [30,31]. Most of the studies were conducted in the USA [18,33,34,35,36]. In addition, the results of the quality assessment using NOS are shown in Table S4. According to quality assessment, two studies had five points [18,35], and the others had six points [33,34,36,37].

**Figure 2:**
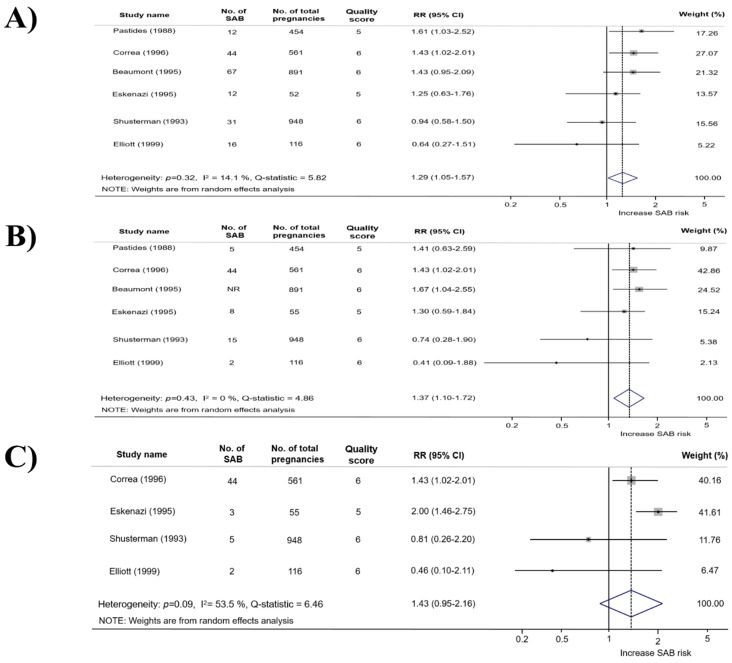
Meta-analysis for cohort and case-controls studies presenting the association between the risk of spontaneous abortion and: Fab work (**A**); photolithography work (**B**); and ethylene glycol ether exposure (**C**).

The pooled effect of total Fab-work and SA is presented as forest plot in the meta-analysis (Figure 2A). Six studies for female workers found that the risk of SA was significantly higher in Fab workers than that in non-Fab workers (RR, 1.29; 95% CI, 1.05–1.57; I^2^, 14.1%). According to the study design (Table 1), cohort studies showed that SA risk in Fab workers was significantly higher than that in non-Fab workers (RR, 1.43; 95% CI, 1.17–1.76; I^2^, 0%). In contrast, case-control studies were not significant (OR, 0.86; 95% CI, 0.57–1.30; I^2^, 0%).

**Figure 3:**
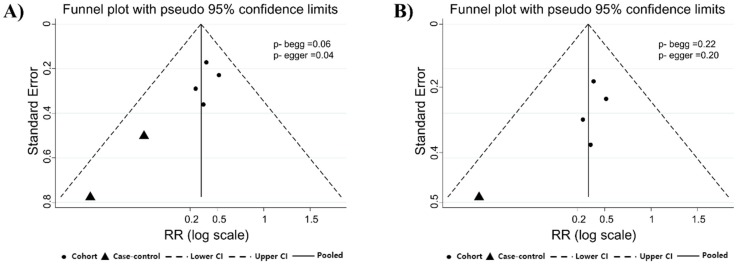
Funnel plot for publication bias in meta-analysis on the association between the risk of spontaneous abortion and photo-lithography work: before sensitivity analysis (**A**); and after sensitivity analysis (**B**).

Many workers are engaged in various processes in the semiconductor industry. Hence, the meta-analysis of the specific processes was conducted. From each study, we were able to identify the processes in which workers were engaged, such as photolithography, etching, masking (photolithography and etching), dopants, thin-film, and diffusion (ion-implant and thin-film). As shown in Figure 2B, we identified information on photolithography from six studies. Although there was a statistically significant association between photolithography work and SA (RR, 1.37; 95% CI, 1.10–1.72, I^2^, 0.0%), publication bias was identified based on the Egger test (*p* = 0.04, Figure 3A). Thus, we used the trim and fill method to adjust for funnel plot asymmetry, but it revealed no trimming was performed and the data were unchanged. After excluding the study that was biased in the funnel plot from meta-analysis, we could not identify statistically significant publication bias and SA risk was still significant (RR, 1.41, 95% CI, 1.13–1.77, I^2^, 0.0%; the Begg test (*p* = 0.22); the Egger test (*p* = 0.20), Figure 3B). According to the study design (Table 1), cohort studies showed that the SA risk in photolithography process was consistently higher than that in non-photolithography process (RR, 1.47; 95% CI, 1.16–1.85; I^2^, 0.0%). In contrast, case-control studies were not significant (OR, 0.63; 95% CI, 0.28–1.41; I^2^, 0%).

In addition, we conducted a meta-analysis, which described possible chemical exposure in the semiconductor industry. As a result, we identified EGE and fluoride exposures, as shown in Figure 2C and Tables S1 and S2. Four studies [33,35,36,37] reported EGE exposure information, and only one study could identify fluorides information for both cohort and case-control studies [35,37]. However, there was no statistically significant association, and there was a high heterogeneity among the studies (EGE; RR, 1.43; 95% CI, 0.95–2.16; I^2^, 53.5%; fluorides; RR, 1.20; 95% CI, 0.73–1.96; I^2^, 0.0%, respectively). Based on the study design, cohort studies showed that the SA risk was higher in the EGE exposure group than that in the non-exposure group, but it had consistently high heterogeneity (RR, 1.70; 95% CI, 1.22–2.36; I^2^, 50.2%). Additionally, case-control studies were not statistically significant (OR, 0.67; 95% CI, 0.28–1.61; I^2^, 0.0%). In the case of fluorides, both cohort and case-control studies were not statistically significant (RR, 1.14; 95% CI, 0.66–1.99; OR, 1.44; 95% CI, 0.49–4.18; respectively). Consequently, we did not find any association between chemical exposures and SA.

## 4. Discussion

Our study is the first systematic review and meta-analysis to identify a positive association between Fab-work, specific process, and chemicals and SA. In the meta-analysis, the participants were semiconductor workers who worked during 1980–1993. Compared with the conventional cohort studies, the four cohort studies did not include the entire cohort of semiconductor workers. These included three retrospective cohort studies and one prospective cohort study that selected pregnant women among all cohorts to identify pregnancy results. However, only 28% (759 women) of the women who participated in the screening study were able to follow-up. We could not generalize the studies to the corresponding semiconductor cohort. In addition, the quality assessment of each study scored five or six points on a 1–9 points scale, and it was difficult to identify these as high-quality studies.

One nested case-control study of semiconductor workers in Korea was published in 2017 [21]. This study was based on women workers who were able to become pregnant and included the entire cohort. In this study, 65% of the full-time workers participated in the survey, 99.9% of the participants (14,226 people) agreed to use personal information, and 2242 women experienced pregnancy. According to the quality assessment, the score was eight points, and it was evaluated as high-quality. Based on exposure data since 1996, no statistically significant association was found between the risk of SA and Fab work from the study. When we considered the study for meta-analysis, it was statistically significant (RR, 1.27; 95% CI, 1.06–1.52). However, as discussed above, there was a significant difference in the working environment and exposure materials between workers in the period 2015 and 1980–1993, thus we did not include this study in the meta-analysis.

Female Fab workers during 1980–1993 had a higher risk (RR, 1.29; 95% CI, 1.05–1.57; I^2^, 14.1%) of SA than non-Fab female workers and this was statistically significant. According to study design, only cohort studies were statistically significant, and there was non-heterogeneity in the meta-analysis (cohort; RR, 1.43; 95% CI, 1.17–1.76; I^2^, 0%; case-control; OR, 0.86; 95% CI, 0.57–1.30; I^2^, 0%; respectively).

Based on the meta-analysis of the specific process, we identified the chemicals used in the processes by referring to the report published by the Korea Occupational Safety and Health Agency [38]. This report presented the chemical materials that could be exposed in the process and indicated hazard based on exposure standards and threshold limit values (TLVs) for mixtures standards. In addition, it represented reproductive toxicity in accordance with the Globally Harmonized System of Classification and Labeling of Chemicals (GHS) standards. We identified chemicals in the semiconductor process which could cause SA. The classification of the reproductive toxicity of GHS is divided into three categories (1A: substances with evidence in humans judged to have adverse effects on sexual function, reproductive ability or development; 1B: a substance that has enough animal test evidence to assume that it has an adverse effect on a person’s sexual function, reproductive ability, or development; and 2: substance with evidence of human or animal test that is suspected to have adverse effects on sexual function, reproductive ability or development in humans) [39]. Materials included in the TLV standards or GHS reproductive toxicity category that are used in photolithography are N,-N-Dimethylacetamide (DMAc, 1B), 2-Ethoxyethanol (1B), Ethylbenzene (1B), 2-Methoxy-1-propanol (β- PGME, 1B, 2-Methoxy-1-propyl acetate (β-PGMEA, 1A), Ethylene glycol (1B), and Xylene (1B). In the case of the diffusion process, nitrous oxide (1A) was identified [38].

However, one of the limitations of this meta-analysis was difficulty in detailed identification of the exposed chemicals. In this study, EGE is considered as chemical exposure material in photolithography. Concerns about chronic exposure to EGE were raised in 1983 [40]. Further, organic solvents of EGE, which are known to have reproductive toxicity, were used in photolithography [34]. At this time, the environmental concentration of EGE was measured, and it was confirmed to be low. In the meta-analysis, the risk of SA in workers in the photolithography group was slightly higher (RR, 1.37; 95% CI, 1.10–1.72, I^2^, 0.0%) than that in workers in office and other processes. According to study design, only cohort studies were statistically significant and there was non-heterogeneity from the meta-analysis. However, it can be estimated as publication bias (Egger test, *p* = 0.04). Through the sensitivity analysis, we could evaluate the association between photolithography work and SA risk without publication bias (the Begg test (*p* = 0.22); the Egger test (*p* = 0.20)). Specifically, EGE exposure meta-analysis had high heterogeneity in both total studies and cohort studies. As a result, we could not find a statistically significant association between specific process, chemical exposures, and SA in the semiconductor industry.

Through systematic review, we searched the literature considering semiconductor work and SA risk in female workers. However, due to the lack of studies, we could not evaluate SA risk based on current working environment. The working periods of the studies included in the meta-analysis ranged from about 1980 to 1993. Due to changes in the working environment, workers exposed to the current environment and workers who worked during 1980–1993 are quite different. Thus, it is necessary to reevaluate the risk of SA in workers by constructing elaborate cohort studies taking into consideration the current environment, especially since most of the workers in the comparison group from the previous studies were office or administrative workers who could have been exposed to low levels of semiconductor-related chemicals. In the case of office workers, supervisors or administrative staffs, their age, education level, income level, and work-beginning period are different from those of Fab workers. Supervisors and administrative staffs may have worked as Fab workers in the past, but their job tenure must be at least a certain length. In addition, the distribution of other confounders such as the first pregnancy age, stress, and working environment may be different. Moreover, a recent case-control study based on semiconductor workers reported that SA risk for packaging process workers whose pregnancy year was prior to 2008 was higher than that for those whose pregnancy year was after 2009. This suggests that further research on SA is needed.

## 5. Conclusions

This study indicates that there is a statistically significant association between Fab-work and SA. Based on the meta-analysis, we can confirm the effects of various processes and chemical exposures on SA, but it needs to be interpreted carefully considering the publication bias. Because of the exposure environment changes due to the rapid change in the semiconductor industry, future studies need to take into account these differences. Therefore, it is necessary to conduct an elaborate cohort study taking into consideration the current environment such as exposure status, working period, pregnancy period, automated system, wafer size, etc. In the future, we expect to clearly assess the risk of SA in semiconductor workers.

## Figures and Tables

**Table 1:** Meta-analysis for studies the association between the risk of spontaneous abortion and Fab work, photolithography work, ethylene glycol ether, and fluorides exposure by study design.

Exposure	First Author (Year)	RR (95% CI)	Design	Summary RR /OR (95% CI)
Fabrication			Total	1.29 (1.05–1.57)
	Pastides (1988)	1.61 (1.03–2.52)	Cohort	1.43 (1.17–1.76)
	Correa (1996)	1.43 (1.02–2.01)		
	Beaumont (1995)	1.43 (0.95–2.09)		
	Eskenazi (1995)	1.25 (0.63–1.76)		
	Shusterman (1993)	0.94 (0.58–1.50)	Case-control	0.86 (0.57–1.30)
	Elliott (1999)	0.64 (0.27–1.51)		
Photo-lithography work			Total	1.37 (1.10–1.72)
			Sensitivity analysis	1.41 (1.13–1.77)
	Pastides (1988)	1.41 (0.63–2.59)	Cohort	1.47 (1.16–1.85)
	Correa (1996)	1.43 (1.02–2.01)		
	Beaumont (1995)	1.67 (1.04–2.55)		
	Eskenazi (1995)	1.30 (0.59–1.84)		
	Shusterman (1993)	0.74 (0.28–1.90)	Case-control	0.63 (0.28–1.41)
	Elliott (1999)	0.41 (0.09–1.88)		
EGE exposure			Total	1.43 (0.95–2.16)
	Correa (1996)	1.43 (1.02–2.01)	Cohort	1.70 (1.22–2.36)
	Eskenazi (1995)	2.00 (1.46–2.75)		
	Shusterman (1993)	0.81 (0.26–2.20)	Case-control	0.67 (0.28–1.61)
	Elliott (1999)	0.46 (0.10–2.11)		
Fluorides exposure			Total	1.20 (0.73–1.96)
	Eskenazi (1995)	1.14 (0.66–1.99)	Cohort	1.14 (0.66–1.99)
	Elliott (1999)	1.44 (0.49–4.18)	Case-control	1.44 (0.49–4.18)

Abbreviation: EGE, Ethylene glycol ether.

## Supplementary Materials

The following are available online at https://www.mdpi.com/1660-4601/16/23/4626/s1, Table S1: Characteristics of individual cohort studies included in the meta-analysis for the association between spontaneous abortion (SA) and semiconductor work among female semiconductor employees, Table S2: Characteristics of individual case-control studies included in the meta-analysis for the association between SA and semiconductor work among female semiconductor employees, Table S3: Characteristics of most recent case-control study, not-included in the meta-analysis for the association between female SA and semiconductor work among female semiconductor employees, Table S4: Quality assessment of studies in systematic review and meta-analysis using the Newcastle-Ottawa Scale.
