# Supplementary material for: Semiconductor Work and the Risk of Spontaneous Abortion: A Systematic Review and Meta-Analysis
Source: Int J Environ Res Public Health. 2019 Nov 21;16(23):4626. doi: 10.3390/ijerph16234626 (PMC6926641; doi:10.3390/ijerph16234626)
Supplement: Supplementary file 1 [file ijerph-16-04626-s001.zip › Supplementary_Semiconductor and Spontanoeus abortion_systematic and meta-analysis_191120.docx]

Table S1. Characteristics of individual cohort studies included in the meta-analysis for the association between spontaneous abortion (SA) and semiconductor work among female semiconductor employees

| First author [Ref.]  (Publication year /  Working periods of subjects) | Design /  Study quality / Remarks | Exposure status | Cohort  N | SAB  N | RR (95% CI) |
| --- | --- | --- | --- | --- | --- |
| Pastides^1^ [18] |  |  |  |  |  |
| 1998 /  Phase I study,  1984;  Phase II study,  1986 | Cohort /  5 /  RRs were adjusted for reporting bias | Non-Exposed  (Un-exposed to process chemicals, including clerical/administrative staff & engineers) | 420 | 93 | 1.00 |
|  |  | Exposed (by calculation)^2^ |  |  | 1.61 (1.03-2.52) ^2^ |
|  |  | Photo-lithography work (exposure to solvents) | 16 | 5 | 1.41 (0.63-2.59) |
|  |  | Diffusion work^4^ (including ion implant and epitaxy) (exposure to toxic gases, acid, and metals) | 18 | 7 | 1.76 (0.90-2.87) |
| Correa^1^ (33) |  |  |  |  |  |
| 1996 /  1980-1989 | Cohort /  6 /  RRs were stratified by number of previous pregnancies, and adjusted for maternal age and education, study plant, prior SAB history and year of conception | Non-Exposed (Un-exposed to EGE) | 332 | 49 | 1.00 |
|  |  | Exposed to EGE (Regarded as photolithography exposure, by calculation)^2^ |  |  | 1.43 (1.02–2.01) ^2^ |
|  |  | Photoresist work (by calculation)^2^ |  |  | 1.92 (0.98-3.78) ^2^ |
|  |  | Low exposure to EGE  (Develop, strip & clean work) | 125 | 20 | 1.00 (0.60-1.70) |
|  |  | Intermediate exposure^3^  (Work on a combination of photoresist and  non-photoresist during critical periods) | 74 | 14 | 1.40 (0.80-2.60) |
|  |  | High exposure^3^  (Photoresist work during critical periods) | 30 | 10 | 2.80 (1.40-5.60) |
| Beaumont (34) |  |  |  |  |  |
| 1995 /  1986-1989 | Cohort /  6 /  RRs were adjusted for age, ethnicity, smoking, SES, pregnancy history, pregnancy start year and workplace stress | Unexposed (Non-Fab office workers) | 444 | 46 | 1.00 |
|  |  | Exposed (Fab workers) | 447 | 67 | 1.43 (0.95–2.09) |
|  |  | Masking |  |  | 1.78 (1.17-2.62) |
|  |  | Photolithography |  |  | 1.67 (1.04-2.55) |
|  |  | Etching |  |  | 2.08 (1.27-3.19) |
|  |  | Dopants & Thin film/Ion implant |  |  | 1.17 (0.68-1.93) |
|  |  | Furnace |  |  | 1.07 (0.57-1.93) |
|  |  | Thin film/Ion implant |  |  | 1.38 (0.70-2.53) |
| Eskenazi (35) |  |  |  |  |  |
| (1995 / 1989-1991) | Cohort /  5 /  RRs were adjusted for risk scores constructed by age, smoking, ethnicity, gravidity, history of SAB, education, and household income | Unexposed (Non-Fab office workers, including non-Fab supervisors & engineers) | 33 | 15 | 1.00 |
|  |  | Exposed (Fab workers, including Fab supervisors &  engineers) | 19 | 12 | 1.25 (0.63–1.76) |
|  |  | Masking | 12 | 8 | 1.30 (0.59-1.84) |
|  |  | Dopants & Thin film/Ion implant | 7 | 5 | 1.39 (0.51-1.96) |
|  |  | Fab supervisors & engineers | 3 | 2 | 1.47 (0.61-3.55) ^4,5^ |
|  |  | Unexposed to EGE & Fluoride | 38 | 19 | 1.00 |
|  |  | EGE exposure | 3 | 3 | 2.00 (1.46-2.75) ^4,5^ |
|  |  | Fluoride exposure | 14 | 8 | 1.14 (0.66-1.99) ^4,5^ |

EGE, ethylene glycol ether; Fab, fabrication

1. Pregnancy spell-based analysis

2. The calculated RR (95% CI) of the exposed was meta-analyzed using two or three exposure groups

3. The critical exposure periods were defined as month of conception for miscarriage and month of conception or 12 months before conception for subfertility.

4. Outcome variable is the presence of SAB at the first pregnancy in this paper; outcome variables in other papers are the presence of SAB in the whole pregnancies.

5. Crude RR

Table S2. Characteristics of individual case-control studies included in the meta-analysis for the association between SA and semiconductor work among female semiconductor employees

| First author [Ref.]  (Publication year /  Working periods of subjects) | Design /  Study quality / Remarks | Exposure status | Cohort  N | SAB  N | RR (95% CI) |
| --- | --- | --- | --- | --- | --- |
| Shusterman [36] |  |  |  |  |  |
| 1993 /  1986-1987 | Case-control /  6 /  ORs were adjusted for age, ethnicity, education, marriage, prior fetal loss, and alcohol consumption | Unexposed  (Not working in electronic and semiconductor work) | 575 | 272 | 1.00 |
|  |  | Exposed (Any electronic production work) | 70 | 31 | 0.94 (0.58-1.50) |
|  |  | Semiconductor Fab work | 37 | 15 | 0.86 (0.44-1.60) |
|  |  | Masking | 33 | 11 | 0.71 (0.33-1.51)^2^ |
|  |  | Photolithography | 20 | 7 | 0.74 (0.28-1.90) |
|  |  | Etching | 13 | 4 | 0.65 (0.18-2.20) |
|  |  | Diffusion, CVD / Epitaxy | 12 | 6 | 1.09 (0.36-3.31)^2^ |
|  |  | Diffusion | 5 | 4 | 1.70 (0.38-7.30) |
|  |  | CVD / Epitaxy | 7 | 2 | 0.60 (0.09-2.70) |
|  |  | All photoresist^1^ | 31 | 15 | 1.01 (0.53-1.94)^2^ |
|  |  | Positive photoresist (Exposure to EGE) ^1^ | 13 | 5 | 0.81 (0.26-2.20) |
|  |  | Negative photoresist^1^ | 5 | 3 | 1.30 (0.25-5.50) |
|  |  | Photoresist developer^1^ | 13 | 7 | 1.10 (0.42-2.90) |
|  |  | Phosphine^1^ | 5 | 3 | 1.30 (0.25-5.50) |
|  |  | Arsine^1^ | 4 | 2 | 1.10 (0.14-6.00) |
|  |  | Arsenic^1^ | 4 | 1 | 0.53 (0.02-4.20) |
|  |  | PC board manufacturing | 4 | 1 | 0.53 (0.02-4.20) |
|  |  | Assembly | 29 | 15 | 1.10 (0.55-2.20) |
|  |  | Stuffing PC boards | 17 | 7 | 0.87 (0.32-2.30) |
|  |  | Soldering | 23 | 14 | 1.30 (0.62-2.70) |
|  |  | Flux removal | 15 | 10 | 1.40 (0.58-3.40) |
|  |  | Encapsulation | 4 | 3 | 1.60 (0.29-7.70) |
|  |  | Contact cleaner sprays^1^ | 5 | 3 | 1.30 (0.25-5.50) |
|  |  | Flux removal solution^1^ | 14 | 10 | 1.50 (0.66-3.40) |
|  |  | Potting compound^1^ | 2 | 3 | 3.20 (0.47-26.8) |
| Elliott^4^ [37] |  |  |  |  |  |
| 1999 /  1987-1992 | Case-control /  6 /  ORs were adjusted for age at conception, smoking, alcohol consumption, lifting, bending & stress during 1-3 months of pregnancy | Non-Fab workers for first trimester (1-12 weeks) | 34 | 19 | 1.00 |
|  |  | Fab workers for first trimester | 44 | 16 | 0.64 (0.27–1.51) |
|  |  | Masking | 30 | 9 | 0.68 (0.32-1.46)^2^ |
|  |  | Photo-lithography | 11 | 2 | 0.41 (0.09-1.88) |
|  |  | Etching | 19 | 7 | 0.80 (0.33-1.97) |
|  |  | Dopants & Thin film/Ion implant | 9 | 5 | 2.00 (0.20-19.93)^2^ |
|  |  | Furnace | 6 | 4 | 0.70 (0.13-3.70) |
|  |  | Thin film/Ion implant | 3 | 1 | 7.42 (0.76-72.0) |
|  |  | Other Fab work | 16 | 5 | 0.56 (0.17-1.84) |
|  |  | EGE exposure | 10 | 2 | 0.46 (0.10-2.11) |
|  |  | Non-EGE photoresist chemicals | 5 | 1 | 0.45 (0.05-3.89) |
|  |  | Fluorides | 12 | 7 | 1.44 (0.49-4.18) |

OR, odds ratio; EGE, ethylene glycol ether; FAB, fabrication

1. Crude OR

2. Generalized estimating equation (GEE) model, adjusted for age, education, BMI, employment duration at pregnancy, and work location

Table S3. Characteristics of most recent case-control study, not-included in the meta-analysis for the association between female SA and semiconductor work among female semiconductor employees

| First author [Ref.]  (Publication year /  Working periods of subjects) | Design /  Study quality / Remarks | Exposure status | Cohort  N | SAB  N | RR (95% CI) |
| --- | --- | --- | --- | --- | --- |
| Kim [21] |  |  |  |  |  |
| 1999 /  Since 1996 | Case-control /  8 /  ORs were adjusted for age at conception, education, BMI, smoking, prior pregnancy outcome, employment duration at pregnancy, year of conception, work location, and shift work  Cohort /  6 /  RRs were stratified by number of previous pregnancies, and adjusted for maternal age and education, study plant, prior SAB history and year of conception | Non-Fab office work | 2,023 | 99 | 1.00 |
|  |  | Fab work | 1,173 | 291 | 1.08 (0.64-1.81) |
|  |  | Assembly work | 312 | 199 | 1.24 (0.72-2.12) |
|  |  | First pregnancy | 2,144 | 98 |  |
|  |  | Non-Fab office work | 1,231 | 6 | 1.00 |
|  |  | Fab work | 721 | 54 | 1.15 (0.46-2.85) |
|  |  | Assembly work | 192 | 38 | 1.34 (0.52-3.41) |
|  |  | Year of conception: <2009^2^ |  |  |  |
|  |  | Non-Fab office work | 139 | 8 | 1.00 |
|  |  | Fab work | 848 | 80 | 1.54 (0.71-3.32) |
|  |  | Assembly work | 505 | 69 | 2.21 (1.01-4.81) |
|  |  | Year of conception: ≥2009^2^ |  |  |  |
|  |  | Non-Fab office work | 212 | 31 | 1.00 |
|  |  | Fab work | 1,466 | 211 | 1.01 (0.69-1.48) |
|  |  | Assembly work | 867 | 130 | 1.05 (0.71-1.55) |
|  |  | Women whose first, last and job held longest were the same^2^ | | | |
|  |  | Non-Fab office work | 226 | 29 | 1.00 |
|  |  | Fab work | 1,959 | 285 | 1.07 (0.71-1.60) |
|  |  | Assembly work | 1,018 | 175 | 1.19 (0.79-1.81) |

Table S4. Quality assessment of studies in systematic review and meta-analysis using the Newcastle-Ottawa Scale

|  | Quality assessment in cohort studies | | |  |
| --- | --- | --- | --- | --- |
|  | Selection  (maximum 4+)^1^ | Comparability  (maximum 2+)^2^ | Outcome  (maximum 3+)^3^ | Scores  (0 – 9) |
| Authors, Year (Ref.) |  |  |  |  |
| Correa et al. 1996 (33) | 3+ | 2+ | 1+ | 6 |
| Beaumont et al. 1995 (34) | 3+ | 2+ | 1+ | 6 |
| Eskenazi et al. 1995 (35) | 2+ | 2+ | 1+ | 5 |
| Pastides et al. 1988 (18) | 2+ | 2+ | 1+ | 5 |
|  | Quality assessment in case-control studies | | |  |
|  | Selection  (maximum 4+)^1^ | Comparability  (maximum 2+)^2^ | Exposure  (maximum 3+)^3^ | Scores  (0 – 9) |
| Authors, Year (reference) |  |  |  |  |
| Eliott et al. 1999 (37) | 3+ | 2+ | 1+ | 6 |
| Shusterman et al. 1993 (36) | 4+ | 2+ | 0 | 6 |
| Kim et al. 2017 (21) | 4+ | 2+ | 2+ | 8 |

1. In cohort studies, selection was assessed by identifying representativeness of exposed cohort, source of non-exposed cohort, ascertainment of exposure data, and demonstration that outcome of interest was not present at start of the study. In case-control studies, adequacy and representativeness of cases, source and definition of controls were included in selection part.
2. Comparability was identified whether studies controlled for the most important factor or any additional factors such age, sex, year of conception or history of pregnancy in the study design or analysis.
3. In cohort studies, source of outcome or ascertainment of exposure, identifying rates of follow-up or non-response rate. In case-control studies, ascertainment of exposure data, similarity with other studies in identifying patients and controls was examined, and non-response rate. In cohort studies, follow-up length was considered.
